# Early versus delayed mobilization for arthroscopic rotator cuff repair (small to large sized tear): a meta-analysis of randomized controlled trials

**DOI:** 10.1186/s12891-023-07075-5

**Published:** 2023-12-04

**Authors:** Ching-Wei Hu, Sung Huang Laurent Tsai, Chien-Hao Chen, Hao-Che Tang, Chun-Yi Su, Eric H. Tischler, Yi-Chiang Yang, Yi-Sheng Chan, Chih-Hao Chiu, Alvin Chao Yu Chen

**Affiliations:** 1 Department of Orthopaedic Surgery, Keelung branch, Bone and Joint Research Center, Chang Gung Memorial Hospital, Chang Gung University, F7, No 222 Mai-King Road, Keelung, Taiwan; 2 Johns Hopkins Bloomberg School of Public Health, Baltimore, MD USA; 3 Department of Orthopaedic Surgery and Rehabilitation Medicine, Downstate Medical Center, State University of New York, 450 Clarkson Ave, Brooklyn, NY 11203 USA; 4 Department of Physical Medicine and Rehabilitation, Taipei Veterans General Hospital, Taipei, Taiwan; 5 Bone and Joint Research Center, Department of Orthopedic Surgery, Chang Gung Memorial Hospital-Linkou & University College of Medicine, Taoyuan City, Taiwan; 6 Comprehensive Sports Medicine Center, Chang Gung Memorial Hospital Taiwan, Taoyuan City, Taiwan; 7 Department of Orthopedic Surgery, Linkou Chang Gung Memorial Hospital, Taoyuan City, Taiwan

**Keywords:** Arthroscopy rotator cuff tear, Early motion, Early rehabilitation, Early mobilization

## Abstract

**Background:**

The timing to start passive or active range of motion (ROM) after arthroscopic rotator cuff repair remains unclear. This systematic review and meta-analysis evaluated early versus delayed passive and active ROM protocols following arthroscopic rotator cuff repair. The aim of this study is to systematically review the literature on the outcomes of early active/passive versus delayed active/passive postoperative arthroscopic rotator cuff repair rehabilitation protocols.

**Methods:**

A systematic review and meta-analysis of randomized controlled trials (RCTs) published up to April 2022 comparing early motion (EM) versus delayed motion (DM) rehabilitation protocols after arthroscopic rotator cuff repair for partial and full-thickness tear was conducted. The primary outcome was range of motion (anterior flexion, external rotation, internal rotation, abduction) and the secondary outcomes were Constant-Murley score (CMS), Simple Shoulder Test Score (SST score) and Visual Analogue Scale (VAS).

**Results:**

Thirteen RCTs with 1,082 patients were included in this study (7 RCTs for early passive motion (EPM) vs. delayed passive motion (DPM) and 7 RCTs for early active motion (EAM) vs. delayed active motion (DAM). Anterior flexion (1.40, 95% confidence interval (CI), 0.55–2.25) and abduction (2.73, 95%CI, 0.74–4.71) were higher in the EPM group compared to DPM. Similarly, EAM showed superiority in anterior flexion (1.57, 95%CI, 0.62–2.52) and external rotation (1.59, 95%CI, 0.36–2.82), compared to DAM. There was no difference between EPM and DPM for external rotation, retear rate, CMS and SST scores. There was no difference between EAM and DAM for retear rate, abduction, CMS and VAS.

**Conclusion:**

EAM and EPM were both associated with superior ROM compared to the DAM and DPM protocols. EAM and EPM were both safe and beneficial to improve ROM after arthroscopic surgery for the patients with small to large sized tears.

**Supplementary Information:**

The online version contains supplementary material available at 10.1186/s12891-023-07075-5.

## Introduction

Rotator cuff tears are the most common causes of shoulder pain and functional limitation [1–3]. When patients report a poor response to first-line non-operative conservative treatment such as physical therapy, non-steroidal anti-inflammatory drugs (NSAIDs), and/or glucocorticoid injection, surgical intervention is indicated [4]. The current clinical practice guidelines of the American Academy of Orthopedic Surgeons (AAOS) show moderate and limited evidence for the use of corticosteroids, hyaluronic acid, and platelet-rich plasma (PRP) interventions. The purposes of surgical repair include: functional improvement, strength endurance, pain relief, and tendon-bone healing [5–7]. Successful rotator cuff repair relies on both the intraoperative fixation as well as postoperative rehabilitation protocols. Historically, immobilization of the shoulder with a sling or brace for up to 6 weeks [8–12] was generally adopted. Also, extra expenditure for sling or brace could be the drawback of this protocol. Although similar outcomes were generally found in clinical comparative studies, there was yet no consensus regarding the detrimental effects of either additional immobilization or early aggressive motion [8, 13–16].

Prior systematic reviews have suggested that early range of motion protocols may reduce postoperative stiffness with improved function, yet result in recurrent tears for large-sized tears [17–19]. It is noteworthy that the majority of reported outcomes only evaluate the passive range of motion [19–21]. To date, limited systematic review exists for postoperative active range of motion protocols.

Recent randomized controlled trials (RCTs) studied the effect of sling versus no sling as an early active motion (EAM) protocol following rotator cuff repair patients [22, 23]; however, reported results were conflicting. Thus, the aim of this systematic review and meta-analysis is to compare the outcomes of early active motion (EAM) versus delayed active motion (DAM) and early passive motion (EPM) versus delayed passive motion (DPM), respectively after rotator cuff repair.

## Methods

### Research protocol and search question

We conducted this study following the Preferred Reporting Items for Systematic Reviews and Meta-Analyses (PRISMA) guidelines. The study protocol of this systematic review and meta-analysis has been registered on PROSPERO with registration number, CRD42022312691. We specified our study question by patients, interventions, comparisons, and outcomes as follows: patients who underwent rotator cuff repair (patient population), whether those who received early mobilization protocol (intervention) versus late mobilization protocol(comparison), which protocol achieved better functional outcomes including range of motion, pain, and retear rates(outcomes).

### Inclusion and exclusion criteria

Studies were eligible if they met the following criteria: (1) Patients who underwent rotator cuff tear repair; (2) were RCTs (Level of Evidence (LOE): 1) or systematic reviews (LOE: 1); 3) English language and 4) reported range of motion, functional score and retear as primary outcomes. Relevant exclusion criteria included: (1) case reports, case series, basic science experiments, and animal or cadaver studies; (2) conference abstracts without full-length papers; (3) irreparable tear, anteroinferior labral lesions (Bankart) or severe glenohumeral osteoarthritis; (4) less than 3 weeks for follow-up; and (5) insufficient data available for analysis. Two reviewers (CWH, SHLT) independently evaluated eligible studies by titles and abstracts and then reviewed the full text of relevant articles for further qualification. All disagreements between reviewers were resolved through discussion to reach consensus, and the senior author (CHC) was consulted if necessary.

### Literature search

A systematic review and meta-analysis of randomized controlled trials (RCTs) published up to April 01, 2022 comparing early motion (EM) versus delayed motion (DM) rehabilitation protocols after arthroscopic rotator cuff tear repair was conducted. The reporting of this study followed the PRISMA [24]. We searched PubMed/MEDLINE, Embase, Cochrane Central Register of Controlled Trials, Scopus, and ISI Web of Science (also ClinicalTrials.gov for unpublished studies) for articles in a systematic approach employing the combination of key-word and medical subject heading (MeSH) for each database without language restriction including: “rotator cuff,” “shoulder arthroscopy,” “supraspinatus tear,” “early rehabilitation,” “early mobilization,” “early motion,” “sling free” or “brace free.” or “immobilization,” “sling,” “brace,” “sling protection,” and “ brace protection” on April 01, 2022. We presented the detailed search strategy in supplementary Table 1. We also searched the reference lists in the included studies to acquire the additional studies.

### Data extraction

Two independent reviewers (CWH, SHLT) extracted all data independently. An electronic piloted form was created for data extraction. Data extracted from the RCTs included authors, year of publication, region of study, study design, patient characteristics, rehabilitation protocols, follow-up duration, and outcome evaluation. The primary outcomes of the study were ROM and retear rate. The secondary outcomes were functional scores (CMS, SST score, and VAS). We defined 3-month follow-up as short-term, 3 to 6 months as mid-term and more than 6 months or the last follow-up as final. Early ROM rehabilitation specified the initiation of active shoulder mobilization or passive shoulder mobilization within 3 weeks postoperatively. Furthermore, all data on outcomes were collected according to the timeline we defined (pre-operative/baseline, short-term, mid-term and final).

### Assessment of risk of bias and quality of evidence

The quality of the included studies was assessed by two independent reviewers (CWH, SHLT). RCTs were evaluated with the ROB2.0 (Cochrane risk-of-bias tool for randomized trials) tools [25]. Grade-Assessment-of-Quality-of-Evidence was done for each outcome following the recommendations from the Grading of Recommendations, Assessments, Development and Evaluation (GRADE) [26].

### Statistical analysis

The Review Manager Software (RevMan Version 5.3, The Cochrane Collaboration, Copenhagen, Denmark) was employed for meta-analysis. Random-effects model was used due to expected clinical heterogeneity. Odds ratios (ORs) with a 95% confidence interval (CI) and mean difference (MD) with 95% CI were utilized for dichotomous outcomes and continuous outcomes, respectively. We tested for heterogeneity with the x^2^ and Higgins I^2^ tests; according to Cochrane guidelines, moderate heterogeneity was considered in the case of I^2^ > 30% or *P* < 0.05. A *P* value < 0.05 was considered statistically significant in all the analyses. Subgroup analysis was done for different tear sizes.

## Results

### Characteristics of included studies

**Fig. 1:**
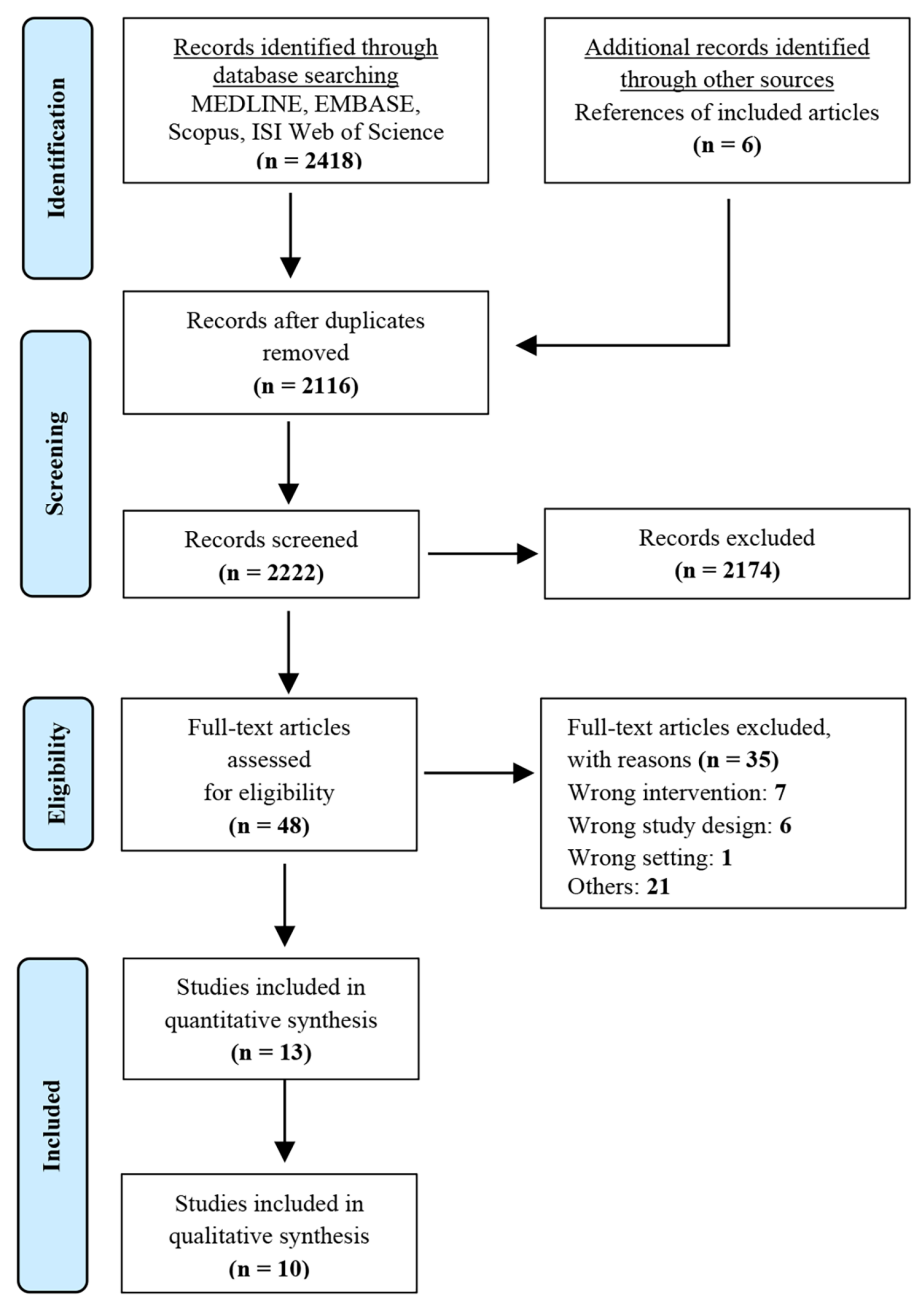
PRISMA flowchart of the study selection criteria. PRISMA, Preferred Reporting Items for Systematic Review and Meta- Analysis

The PRISMA study flow chart is displayed in Fig. 1. Five additional articles were extracted from the reference list of other studies. 2116 articles were identified after removing duplicates. Thirteen RCTs [8, 13–15, 22, 23, 27–33] with 1406 patients conformed to our inclusion criteria and were included in this study (Table 1). Among them, 7 RCTs [8, 13–15, 27–29] met the criteria for early passive motion (EPM) vs. delayed passive motion (DPM) and 7 RCTs [8, 13–15, 27–29] for early active motion (EAM) vs. delayed active motion (DAM). A total of 331 patients (*n* = 331 shoulders) were EPM and 292 patients (*n* = 292 shoulders) were DPM. The mean age ranged from 54.5 to 64.6 years in the EPM group and 55.2 to 65.1 years in the DPM group. 52.65% (*n* = 328) were female. A total of 416 patients were EAM and 407 patients were DAM. The mean age ranged from 52.32 to 57.68 years in the EAM group and 50.43 to 57.2 years in the DAM group. 43.25% (*n* = 356) were female. The final follow-up timing in the included studies ranged from 4 to 24 months (most of them are 12 months). Risk of bias assessment was presented in supplementary Fig. 1 and publication bias was assessed with funnel plots in supplementary Fig. 2 ~ 7 for each outcome. Grade-Assessment-of-Quality-of-Evidence was presented in supplementary Table 2.

**Table 1:** Patient Characteristics in the Included Studies

Study	LOE	Country	Study design	Patient (male/ female)	Tear size (cm)/ Surgical technique	Mean age (range/SD)	Immobilized device/ (removal timing of EM, DM group)	Rehabilitation protocol		Image method/ follow-up	Outcome evaluation
								Early motion timing)	Delayed motion (timing)		
Arndt 2012 [2]	1	France	RCT	92 (34/58)	Partial and full-thickness tear (NA)/ Single or double row	55.3	Sling/ (POW 6, POW 6)	^*^EPM (POD 1)	^*^DPM (POW 6)	CT/ 3, 6, 12, > 12 months	Constant score/ forward flexion / external rotation/ retear rate
Cuff 2012 [11]	1	USA	RCT	68 (38/30)	full-thickness tear/ Transosseousequivalent suture-bridge technique	63.2	Shoulder immobilizer / (POW 6, POW 6)	EPM (POD 2) EAM (POW 6)	DPM (POW 6) DAM (POW 7)	Ultrasound /6, 12 months	ASES score/ SST/ forward flexion/ external rotation/ internal rotation/ cuff healing rate
Kim 2012 [23]	1	South Korea	RCT	105 (44/61)	full-thickness tear (< 3)/Single or double row or suture bridge	60	Brace/ (POW4 ~ 5, POW4 ~ 5)	^#^EPM (POD 1)	^#^DPM (POW 4 ~ 5)	Ultrasound, CT, MRI/ 3, 6, 12 months	VAS/ constant score/ SST/ ASES/ forward flexion/ external rotation/ Internal rotation/ detachment rate
Lee 2012 [28]	1	South Korea	RCT	64 (41/23)	Full-thickness tear (1–5)/ Single row	54.87 (39–66)	Sling/ (POW 6, POW 6)	^*^EPM (POD 1)	*^%^DPM (POW 3)	MRI/ 3, 6, 12 months	VAS/ UCLAS/ active strength/ forward flexion/ external rotation/ retear rate/healing rate
Düzgün 2014 [13]	1	Turkey	RCT	40 (6/34)	Full-thickness tear (1–5)/ Side-to-side repair	57.43 (8.97)	N/A	EPM (POW2) EAM (POW 3)	DPM (POW4) DAM (POW 6)	NA/ 1, 3, 5, 8, 12, 16, 24 months	Active Elevation / forward flexion/ external rotation/ internal rotation
Keener 2014 [22]	1	USA	RCT	124 (73/51)	Full-thickness tear (< 3) / Double row	< 65	Sling/ (POW 6, POW 6)	EPM (POW 1) EAM (POW 6)	DPM (POW 6) DAM (POW 12)	Ultrasound/ 6, 12, > 12 months	VAS/ ASES/ SST/ constant score/ abduction strength/ external rotation strength/healing and retear rate
De Roo 2015 [12]	1	Belgium	RCT	130 (59/71)	Full-thickness tear (< 5)/ Single or double Row	64.8 (9.85)	Brace/ (POW 6, POW 6)	EPM (POD 1) EAM (POW 5)	DPM (POW 5) DAM (POW 6)	Ultrasound/ 6 weeks,4 months	SST, SPADI, constant score/ UCLAS/ active strength/ forward flexion/ external rotation
Sheps 2015 [42]	1	Canada	RCT	189 (115/74)	Full-thickness tear (< 5)/ mini-open rotator cuff repair	55.16 (35–86)	Sling/ (as needed, POW 6)	EAM (POD 1)	DAM (POW 6)	Ultrasound, MRI/ 6 weeks, 3, 6, 12, 24 months	VAS/ forward flexion/ external rotation/ internal rotation/abduction/ Scapular plane elevation
Mazzocca 2017 [35]	1	USA	RCT	58 (40/18)	Full-thickness tear (NA)/ Transosseouse quivalent suture-bridge technique	54.53 (7.5)	Sling/ (POW 6, POW 6)	EAM (POD 2)	DAM (POW 5)	MRI/ 1, 3, 6, 12, 24 weeks	VAS/ WORC/ ASES/SST/SANE/ constant score/ forward flexion/ external rotation/ failure rate
Zhang 2017 [47]	1	China	RCT	132 (69/63)	Full-thickness tear (3–5)/NA	51.38 (11.84)	Brackets/ (POW 6, POW 6)	EAM (POD 3)	DAM (POW 6)	X-ray/ 3, 6, 12 months	VAS/ UCLAS/ constant score/ forward flexion/ external rotation/ re-tear rate
Jenssen 2018 [19]	1	Norway	RCT	118 (69/49)	Full-thickness tear (< 3) / Single row	55.5 (34–73)	Sling, brace/ (POW3, POW6)	^#^EAM (POW3)	^#^DAM (POW 6)	MRI/ 6 weeks, 3, 6, 12 months	VAS for satisfaction/ forward flexion/ external rotation/abduction /healing/ atrophy/fatty infiltration/ WORC/ constant score
Tirefort 2019 [44]	1	Switzerland	RCT	80 (37/43)	Full-thickness tear (< 3)/double row	54.1 (27–78/9.87)	Sling/ (NA, POW 5)	EPM (POD 1) EAM (POD 1)	DPM (POD1) DAM (POW 5)	Ultrasound / 10 days, 1.5, 3, 6 months	VAS/ SANE/ ASES/ forward flexion/ external rotation
Sheps 2019 [43]	1	Canada	RCT	206 (131/75)	Full-thickness tear (1–5)/ single row or double row/ Transosseouse	55.9 (26–79)	Sling/ (as needed, POW 6)	EAM (POD 1)	DAM (POW 6)	Ultrasound/ 6 weeks, 3, 6, 12, 24 months	VAS/ forward flexion/ external rotation/ internal rotation/abduction/ scaption/ strength/ WORC/ SF-36 Scores

RCT: randomized control trial; POD: postoperative day; POW: postoperative week; EM: early motion; DM: delayed motion; EAM: early active motion or early active-assist motion; DAM: delayed active motion; EPM: early passive motion; DPM: delayed passive motion; ASES: American Shoulder and Elbow Surgeons Score; SST: Simple Shoulder Test; UCLAS: University of California at Los Angeles Score; SPADI: Shoulder Pain And Disability Index; WORC: Western Ontario Rotator Cuff index; SANE: Single Assessment Numeric Evaluation; DASH: Disabilities of The Arm Shoulder and Hand; SF-36 Scores: 36-Item Short Form

+: median; *: active motion start POW 6; #: active motion start after removal of sling/brace; %: anterior elevation only and external rotation start from POW 3

### Range of motion

**Fig. 2:**
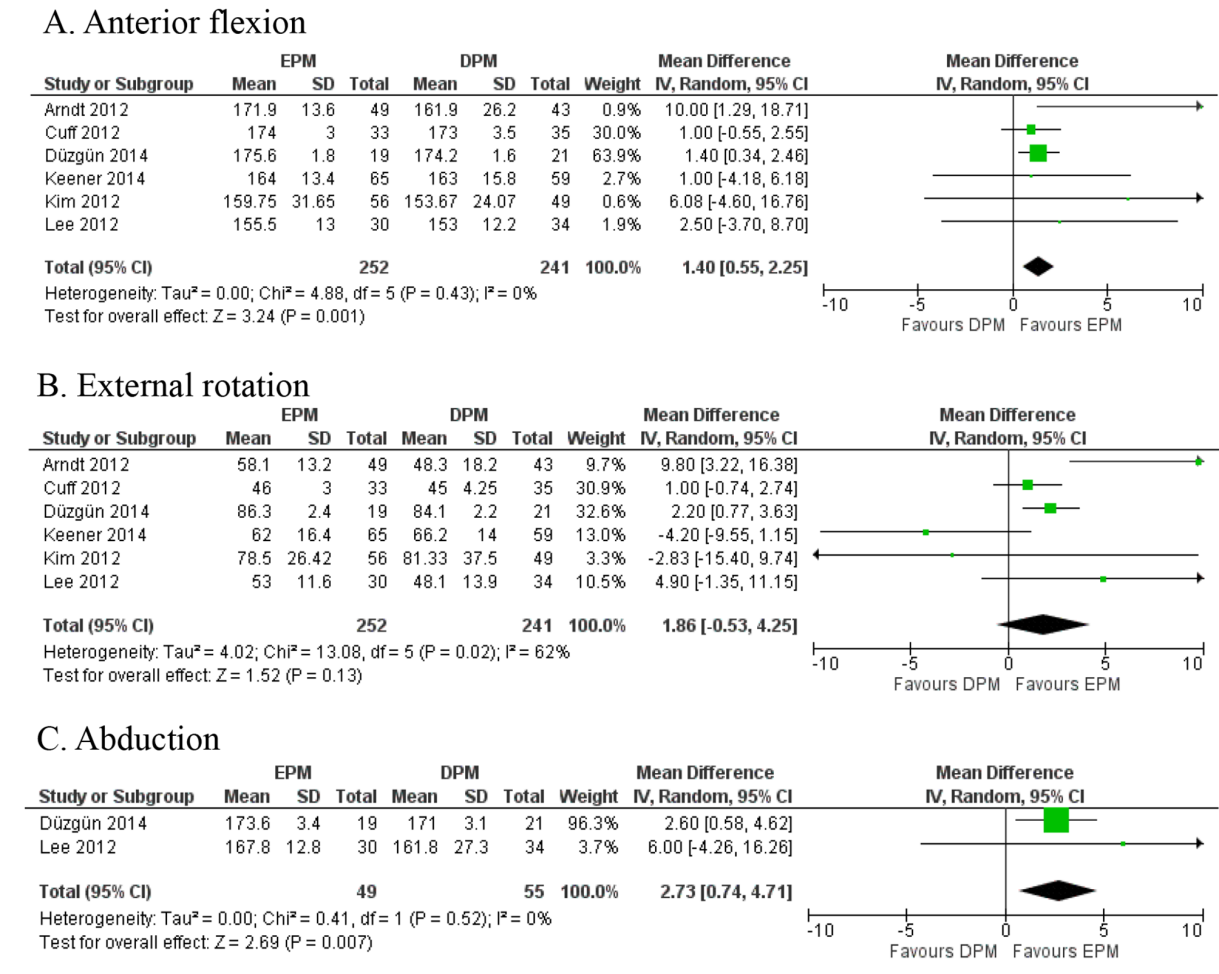
Delayed passive motion (DPM) vs. early passive motion (EPM), ROM including **(A)** Anterior flexion, **(B)** External rotation, **(C)** Abduction, forest plots based on range of motion with random-effects model

**Fig. 3:**
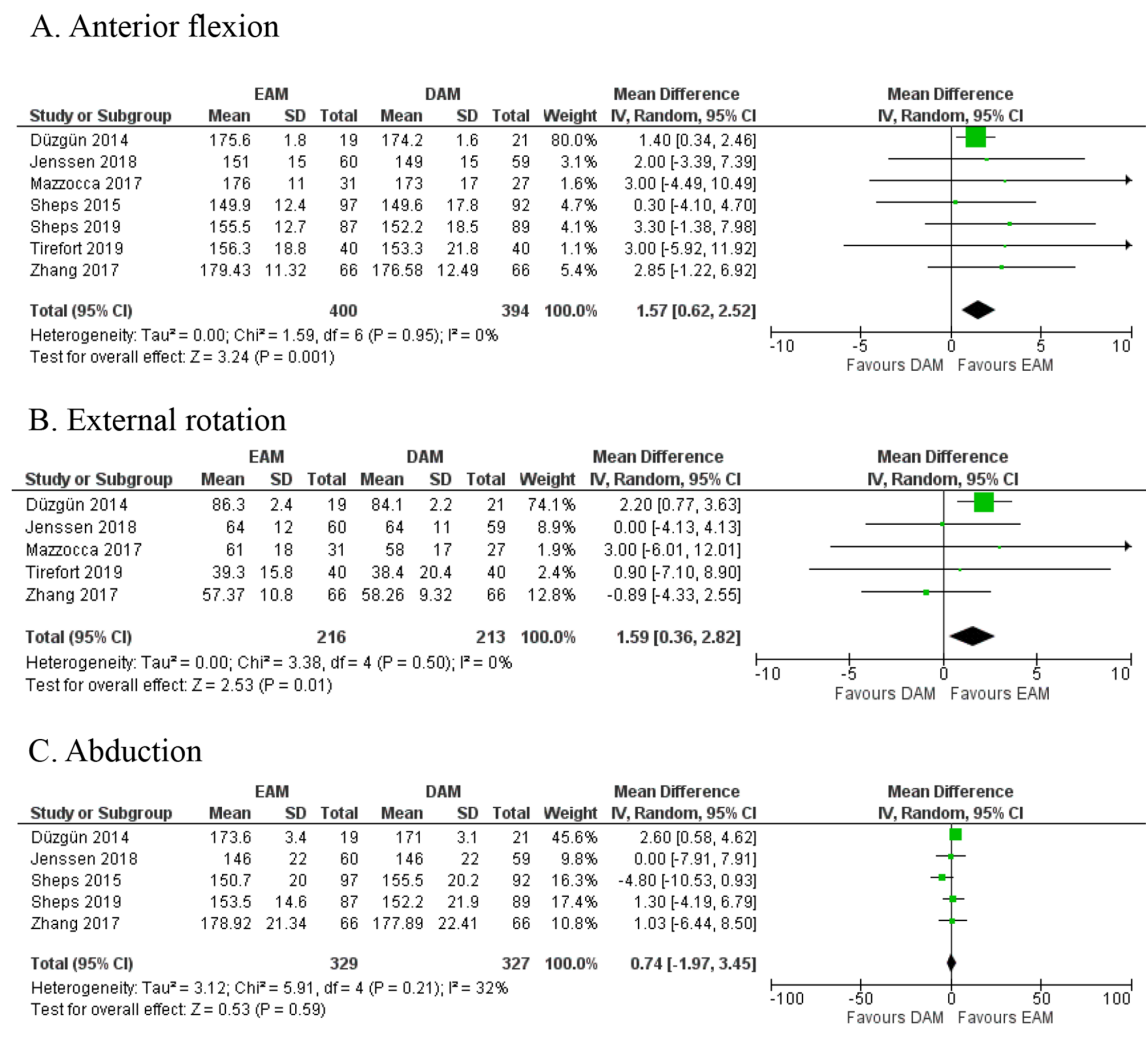
Delayed active motion (DAM) vs. early active motion (EAM), ROM including **(A)** Anterior flexion, **(B)** External rotation, **(C)** Abduction, forest plots based on range of motion with random-effects model

6 studies [8, 13–15, 27, 29] with 493 patients reported this outcome for final follow-up. EPM group showed better anterior flexion (MD 1.40, 95%CI, 0.55–2.25, *p* = 0.01, Quality of Evidence: Moderate) (Fig. 2A) compared to DPM, and there was no statistically significant difference for external rotation (MD 1.86, 95% CI, -0.53-4.25, *p* = 0.13, Quality of Evidence: Moderate) (Fig. 2B). Besides, two studies [13, 29] with 104 patients showed better abduction in EPM (MD 2.73, 95% CI, 0.74–4.71, *p* = 0.007, Quality of Evidence: Moderate) compared to DPM (Fig. 2C). On the other hand, EAM appeared to have better anterior flexion (MD 1.57, 95%CI, 0.62–2.52, *p* = 0.001, Quality of Evidence: Moderate) and external rotation (MD 1.59, 95%CI, 0.36–2.82, *p* = 0.01, Quality of Evidence: High) (Fig. 3A) (Fig. 3B). There was no statistically significant difference for abduction (MD 0.74, 95% CI, -1.97-3.45, *p* = 0.59, Quality of Evidence: High) (Fig. 3C). Additional follow-up outcomes were presented in supplementary Fig. 8 ~ 10, and 12 ~ 15.

### Retear rate

**Fig. 4:**
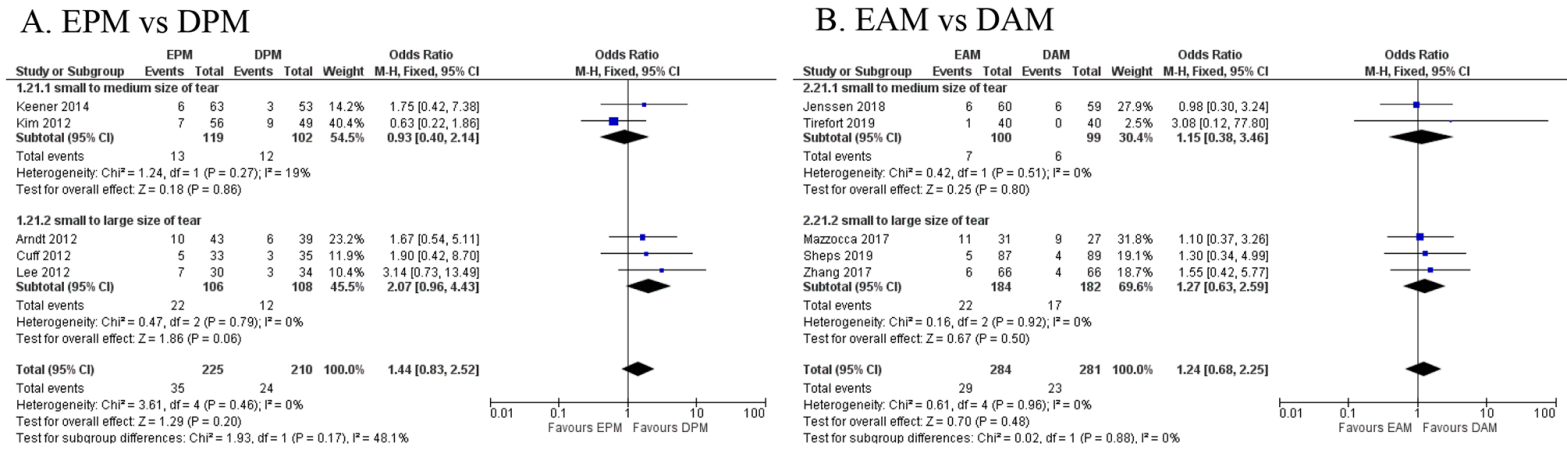
Early passive/active motion (EPM/EAM) vs. delayed passive/active motion (DPM/DAM), forest plots based on retear rate with random-effects model

Compared to DPM/DAM, EPM/EAM demonstrated no significant difference in retear rate with mean difference 1.44 (95% CI, 0.83–2.52, *p* = 0.17, Quality of Evidence: Moderate) / 1.24 (95% CI, 0.68–2.25, *p* = 0.88, Quality of Evidence: High) in 5 studies [8, 13–15, 27] with 435 patients/ 5 studies [22, 23, 30, 32, 33] with 565 patients respectively (Fig. 4).

### Functional score (CMS and SST)

Three studies [8, 15, 27] with 321 patients showed no statistically significant difference in CMS (1.65, 95% CI, -3.03, 6.34, *p* = 0.49) (supplementary Fig. 11.2) between EPM and DPM. Similarly, there was no statistically significant difference in the Simple Shoulder Test (MD 0.35, 95% CI, -0.30, 1.00, *p* = 0.029) (supplementary Fig. 11.3). On the other hand, EAM appeared to have better CMS compared to DAM (2.30, 95% CI, -2.46, 7.06, *p* = 0.34) (supplementary Fig. 16.1) [30, 32, 33]. Additional follow-up outcomes were presented in supplementary Fig. 11.

### Visual analogue scale (VAS)

Five studies [22, 23, 30–32] with 635 patients reported this outcome for final follow-up. There was no statistically significant difference in VAS score between EAM and DAM (MD -0.17, 95% CI, -0.44-0.11, *p* = 0.24) (supplementary Fig. 16.5). Additional follow-up outcomes were presented in supplementary Fig. 16.

### Subgroup analysis

The sub-group analysis was performed to evaluate if large-sized tears are a risk factor for re-tears. Two studies [8, 15] for small to medium size of tear from passive motion protocols were excluded, EPM demonstrated no significant difference in retear rate compared to DPM with mean difference 2.07 (95% CI, 0.96–4.43, *p* = 0.06, Quality of Evidence: Moderate). By excluding two studies [23, 33] for small to medium size of tear from active motion protocols, EAM demonstrated no significant difference in retear rate compared to DAM with mean difference 1.27 (95% CI, 0.63–2.59, *p* = 0.5, Quality of Evidence: High) (Fig. 4).

## Discussion

This systematic review and meta-analysis of RCTs comparing early mobilization in patients receiving rotator cuff repair with those receiving delayed mobilization protocol demonstrated no difference in SST score, CMS, VAS and retear rate.

Recently published meta-analyses [19–21, 34–37] revealed that early mobilization protocols improved postoperative shoulder stiffness and ROM. Unlike prior meta-analyses of RCTs [19–21, 35, 36], our study is the first systematic review to define rehabilitation into active or passive protocols to further explore conflicting results of EAM versus DAM protocol. We reported that EPM and EAM protocol demonstrated significant improvement in degrees of shoulder anterior flexion at final follow-up compared to DPM and DAM and that EAM showed better improvement in external rotation than DAM. However, EPM protocol did not show superiority in external rotation for final follow-up, though improved external rotation was observed at short- and mid-term follow-ups compared to DPM (supplementary Fig. 9). Similar to Li et al [21], we speculated the inconsistency between anterior flexion and external rotation may result from restriction of ROM from rehabilitation protocol which allowed anterior flexion up to 90 degrees, whereas 30 degrees for external rotation in case of excessive loading in most RCTs. On the other hand, there was no strict restriction of ROM for most active motion protocols, which may lead to the superiority of early active motion over delayed in external rotation. This result provides evidence to support the clinical benefits that early active ROM improves postoperative shoulder stiffness for patients receiving rotator cuff repair. As for abduction, there was no difference between EAM and DAM observed at short-term, mid-term and final follow up in our result (Fig. 3, supplementary Fig. 15) and early postoperative pain induced by abduction may be the reason for patients with EAM protocols to avoid abduction, which led to barely satisfactory improvement of abduction for all follow-ups compared to DAM. In fact, postoperative pain is the main reason for restricting initiation of abduction during phase 1 (within 6 weeks after surgery) of rehabilitation [38]. On the other hand, EPM has shown superiority over DPM with abduction, but the limited study amount is the main drawback. More research for abduction would be necessary for better evaluation of postoperative shoulder stiffness.

In addition to postoperative ROM, retear complications following arthroscopic rotator cuff repair remain a major concern for early motion protocols [8, 14, 15, 27]. Our findings indicate that EAM and EPM have no statistically significant retear risk with greater improvement in ROM compared to delayed protocols. These findings are supported by previous studies that demonstrated early motion protocols prevent postoperative shoulder stiffness in patients after rotator cuff repair [19, 21]. Additionally, early motion promotes the circulation of blood and lymph, decreasing the risk for adhesion formation [15, 32]. Tear size has been shown to be a major predictor of rotator cuff repair failure. Large-sized tears have been consistently reported to have a higher retear rate [39–42]. Unfortunately, most of the studies we enrolled in were either all-size tears without differentiating any tear size or small to medium-size tear, which led to difficulties in evaluating the retear rate of large-sized tears. To account for this limitation, we performed a subgroup analysis by excluding four studies [8, 15, 23, 33]. which included only small or medium-size of tear. Our finding pointed out that retear rates were higher among patients with large-sized tears but without statistically significant difference between early versus delayed mobilization protocol (Fig. 4A) (Fig. 4B). Multiple risk factors for retear were reported by many cohort studies and meta-analyses such as preoperative clinical evaluation factors (age, obesity, muscle fatty infiltration, and bone mineral density) and anatomic factors (tear length, width, and amount of retraction), etc [39–47]. Thus, early active motion protocol or no sling protocol [23] may be considered as postoperative rehabilitation for better improvement of ROM if patients have no other risk factor for retear and patients with large size of tear and multiple risk factors may receive delayed motion protocol (postoperative 4 ~ 6 weeks) as postoperative rehabilitation due to concern of retear. However, more studies in the future are necessary for further confirmation of the assumption due to the lack of eligible trails for analysis. When it comes to early motion protocol, we recommend to start pain-free active motion on postoperative day 1 with sling or brace as needed during first postoperative 6 weeks similar to previous study [22] and active overhead motion may be restricted for 12 weeks for concern of retear [48].

Colasanti et al [49] focused on comparison of different surgical techniques and reported that transosseous-equivalent/suture bridge technique and late motion protocol have the highest functional outcomes and lowest retear rate. Unfortunately, there was no further subgroup analysis for different tear sizes and active/passive motion protocols, which may lead to the inconsistency of results from their study. Besides, recent surgical techniques have shown better results in reducing pain after surgeries [50–52] and postoperative multimodal analgesics have been effective [53]. Therefore, most studies showed no statistically significant differences in VAS between the two protocols at the final follow-up, which is consistent with our report.

When it comes to functional scores, Cronin, et al [54] reported the University of California at Los Angeles (UCLA) score to be the most responsive followed by the Adjusted CMS for patients with shoulder arthroplasty but limited studies with the UCLA score and the Adjusted Constant score outcomes were available in our included studies for further analysis. Therefore, CMS and SST were used for further evaluation owing to more reported data. In fact, CMS has been widely used as a commonly reported outcome scale with subjective and objective evaluations such as pain and ROM, respectively. The advantages of CMS are established population normative value which is helpful in further score interpretation [55] and greater weighting of ROM might attribute postoperative evaluation for rotator cuff repair [56]. However, the potential reduced reliability of CMS might occur due to inconsistency between patient assessment and objective shoulder measurements [56], which may be the explanation for our outcome that EAM improved ROM with insignificant improvement in CMS. SST consists 12 questions with yes/no responses and it is faster and easier to complete compared to other functional tests in clinical practice. Though, simplified questions with restricted total points might lead to neglect of clinically significant differences.

Although this study can evaluate passive versus active in the early and delayed postoperative rehabilitation settings, there are several limitations in this study. First, rehabilitation protocol variation among included studies may lead to heterogeneity that affects the final analysis validity. Additionally, follow-up timing protocols varied among studies; however, this was accounted for by clear definitions of short-term, mid-term and final time periods. Some studies [19, 35, 57] suggested that better improvement of ROM from early rehabilitation protocols may fade away over time and the optimal follow-up time point may be 1.5 ~ 2 years in order to reveal complete long-term postoperative outcomes. Due to limited studies providing reported outcomes for long-term follow-up (up to two years) leading to insufficient long-term evaluation, more studies for longer follow-up time are necessary in the future. Second, as for tear characteristics, limited studies provided complete information such as tear-size, length, width, fatty infiltration status, and perioperative medication use. Surgical techniques may also be a confounding factor to the results, given most studies did not provide details of their surgical technique in their trials. Third, minimal clinically important difference (MCID) of ROM and functional score for shoulder after arthroscopic rotator cuff repair has not been well established yet. Thus, statistically significant differences in our results may not indicate clinical difference. Lastly, as seen in many other surgically oriented randomized controlled trials, the double-blinded process for patients and outcome assessors is difficult to achieve, which might result in biases in our analysis.

## Conclusion

EAM and EPM were both associated with superior ROM especially for anterior flexion, compared to the DAM and DPM protocol with similar clinical results. Patients with small to large-sized tears may be permitted to start early mobilization after arthroscopic surgery and further long-term studies are necessary to establish its superiority in patients’ mobility performance.

## Electronic supplementary material

Below is the link to the electronic supplementary material.

**Supplementary Material 1**

**Supplementary Material 2:** Table 1: Electronic search strategy. Four databases (Medline, Embase, Web of Science, Scopus) were searched systematically with keywords specified by patients, interventions, comparisons, and outcomes as follows: patients who underwent rotator cuff repair (patient population), whether those who received early mobilization protocol (intervention) versus late mobilization protocol(comparison), which protocol achieved better functional outcomes including range of motion, pain, and retear rates(outcomes). Figure 1: Risk of bias summary and risk of bias graph. ROB2.0 (Cochrane risk-of-bias tool for randomized trials) tools were used to evaluate the risk of bias from each included study judged by 7 domains (Random sequence generation, allocation concealment, blinding outcomes assessment, incomplete outcome data, selective reporting, and other bias). Red: serious concern; yellow: unclear; Green: no concern. Figures 2–7: Funnel plot of outcomes. Publication bias was assessed with funnel plots for each outcome (EPM vs DPM: ROM, functional scores and re-tear rate; EAM vs DAM: ROM, functional scores and re-tear rate). Figures 8–16: Forest plot of other outcomes. Forest plot with random-effects model for delayed passive/active motion (DPM/DAM) vs early passive/active motion (EPM/DPM) of each outcome (ROM, functional scores, and re-tear rate) at different periods (preoperative, short-term, and mid-term). Table 2: Grade-Assessment-of-Quality-of-Evidence. Grade-Assessment-of-Quality-of-Evidence for each outcome (ROM, functional scores, and re-tear rate) following the recommendations from the Grading of Recommendations, Assessments, Development, and Evaluation. (Confidence level: Very low: very little confidence in the estimate of the effect and the true effect is likely to be different from estimate; Low: the estimate of the effect is limited and the true effect may be different from the estimate; Moderate: moderate confidence in the estimate of the effect and the true effect is likely to be close to estimate; High: very confident that the true effect is close to the estimate.)

## Data Availability

The datasets used and/or analyzed in this study are available from the corresponding author on reasonable request.
