# Correction: Early versus delayed mobilization for arthroscopic rotator cuff repair (small to large sized tear): a meta-analysis of randomized controlled trials

**DOI:** 10.1186/s12891-024-08079-5

**Published:** 2024-11-28

**Authors:** Ching-Wei Hu, Sung Huang Laurent Tsai, Chien-Hao Chen, Hao-Che Tang, Chun-Yi Su, Eric H. Tischler, Yi-Chiang Yang, Yi-Sheng Chan, Chih-Hao Chiu, Alvin Chao Yu Chen

**Affiliations:** 1 Department of Orthopaedic Surgery, Keelung Branch, Bone and Joint Research Center, Chang Gung Memorial Hospital, Chang Gung University, F7, No 222 Mai-King Road, Keelung, Taiwan; 2 Johns Hopkins Bloomberg School of Public Health, Baltimore, MD USA; 3 Department of Orthopaedic Surgery and Rehabilitation Medicine, Downstate Medical Center, State University of New York, 450 Clarkson Ave, Brooklyn, NY 11203 USA; 4 Department of Physical Medicine and Rehabilitation, Taipei Veterans General Hospital, Taipei, Taiwan; 5 Bone and Joint Research Center, Department of Orthopedic Surgery, Chang Gung Memorial Hospital-Linkou & University College of Medicine, Taoyuan City, Taiwan; 6 Comprehensive Sports Medicine Center, Chang Gung Memorial Hospital Taiwan, Taoyuan City, Taiwan; 7 Department of Orthopedic Surgery, Linkou Chang Gung Memorial Hospital, Taoyuan City, Taiwan

**Correction** **:** **BMC Musculoskelet Disord 24, 938 (2023)**

**https://doi.org/10.1186/s12891-023-07075-5**

Following publication of the original article [1], the authors identified errors in the names of Sung Huang Laurent Tsai and Ching-Wei Hu.

The names were corrected from

Sung Laurent Huang Tsai and Hu Ching-Wei

to

Sung Huang Laurent Tsai and Ching-Wei Hu.

The author group has been updated above and the original article [1] has been corrected.
