# Supplementary material for: Early versus delayed mobilization for arthroscopic rotator cuff repair (small to large sized tear): a meta-analysis of randomized controlled trials
Source: BMC Musculoskelet Disord. 2023 Dec 4;24:938. doi: 10.1186/s12891-023-07075-5 (PMC10694899; doi:10.1186/s12891-023-07075-5)
Supplement: Supplementary file 2 — Supplementary Material 2: Table 1: Electronic search strategy. Four databases (Medline, Embase, Web of Science, Scopus) were searched systematically with keywords specified by patients, interventions, comparisons, and outcomes as follows: patients who underwent rotator cuff repair (patient population), whether those who received early mobilization protocol (intervention) versus late mobilization protocol(comparison), which protocol achieved better functional outcomes including range of motion, pain, and retear rates(outcomes). Figure 1: Risk of bias summary and risk of bias graph. ROB2.0 (Cochrane risk-of-bias tool for randomized trials) tools were used to evaluate the risk of bias from each included study judged by 7 domains (Random sequence generation, allocation concealment, blinding outcomes assessment, incomplete outcome data, selective reporting, and other bias). Red: serious concern; yellow: unclear; Green: no concern. Figures 2–7: Funnel plot of outcomes. Publication bias was assessed with funnel plots for each outcome (EPM vs DPM: ROM, functional scores and re-tear rate; EAM vs DAM: ROM, functional scores and re-tear rate). Figures 8–16: Forest plot of other outcomes. Forest plot with random-effects model for delayed passive/active motion (DPM/DAM) vs early passive/active motion (EPM/DPM) of each outcome (ROM, functional scores, and re-tear rate) at different periods (preoperative, short-term, and mid-term). Table 2: Grade-Assessment-of-Quality-of-Evidence. Grade-Assessment-of-Quality-of-Evidence for each outcome (ROM, functional scores, and re-tear rate) following the recommendations from the Grading of Recommendations, Assessments, Development, and Evaluation. (Confidence level: Very low: very little confidence in the estimate of the effect and the true effect is likely to be different from estimate; Low: the estimate of the effect is limited and the true effect may be different from the estimate; Moderate: moderate confidence in the estimate of the effec [file 12891_2023_7075_MOESM2_ESM.docx]

**SUPPLEMENTARY CONTENT**

**The following material accompanies the article “Early versus delayed mobilization for arthroscopic rotator cuff repair (small to large sized tear): A meta-analysis of randomized controlled trials”**

**Table of Content**

**Table 1: Electronic search strategy p. 3-5**

Four databases (Medline, Embase, Web of Science, Scopus) were searched systematically with keywords specified by patients, interventions, comparisons, and outcomes as follows: patients who underwent rotator cuff repair (patient population), whether those who received early mobilization protocol (intervention) versus late mobilization protocol(comparison), which protocol achieved better functional outcomes including range of motion, pain, and retear rates(outcomes).

**Figure 1: Risk of bias summary and risk of bias graph p. 6**

ROB2.0 (Cochrane risk-of-bias tool for randomized trials) tools were used to evaluate the risk of bias from each included study judged by 7 domains (Random sequence generation, allocation concealment, blinding outcomes assessment, incomplete outcome data, selective reporting, and other bias). Red: serious concern; yellow: unclear; Green: no concern.

**Figure 2~7: Funnel plot of outcomes p. 7-12**

Publication bias was assessed with funnel plots for each outcome (EPM vs DPM: ROM, functional scores and re-tear rate; EAM vs DAM: ROM, functional scores and re-tear rate)

**Figure 8~16: Forest plot of other outcomes p. 13-18**

Forest plot with random-effects model for delayed passive/active motion (DPM/DAM) vs early passive/active motion (EPM/DPM) of each outcome (ROM, functional scores, and re-tear rate) at different periods (preoperative, short-term, and mid-term)

**Table 2: Grade-Assessment-of-Quality-of-Evidence p. 19-20**

Grade-Assessment-of-Quality-of-Evidence for each outcome (ROM, functional scores, and re-tear rate) following the recommendations from the Grading of Recommendations, Assessments, Development, and Evaluation. (Confidence level: Very low: very little confidence in the estimate of the effect and the true effect is likely to be different from estimate; Low: the estimate of the effect is limited and the true effect may be different from the estimate; Moderate: moderate confidence in the estimate of the effect and the true effect is likely to be close to estimate; High: very confident that the true effect is close to the estimate.)

**Supplementary Table 1: Electronic search strategy**

20210401

Abbreviations: AF: all fields; exp: exploded; lim: limitation

MEDLINE (1152udies)

1. rotator cuff. AF
2. shoulder arthroscopy. AF
3. supraspinatus tear. AF
4. subscapularis tear. AF
5. biceps tendon tear. AF
6. shoulder instability. AF
7. 1 or 2 or 3 or 4 or 5 or 6
8. early rehabilitation. AF
9. early mobilization. AF
10. early motion. AF
11. rehabilitation. AF
12. mobilization. AF
13. motion. AF
14. sling free. AF
15. brace free. AF
16. 8 or 9 or 10 or 11 or 12 or 13 or 14 or 15
17. sling. AF
18. brace. AF
19. sling protection. AF
20. brace protection. AF
21. immobilization. AF
22. 17 or 18 or 19or 20 or 21
23. 7 and (16 or 22)
24. Observational Study
25. Clinical trial
26. Randomized Controlled Trial
27. Systematic review
28. Meta-analysis
29. 24 or 25 or 26 or 27 or 28
30. 23 and 29

EMBASE (synonyms were included in each search) (725 studies)

1. rotator cuff injury /exp
2. shoulder arthroscopy /exp
3. supraspinatus tear /exp
4. subscapularis tear /exp
5. biceps tendon tear/exp
6. recurrent shoulder dislocation /exp
7. 1 or 2 or 3 or 4 or 5 or 6
8. rehabilitation /exp
9. mobilization /exp
10. motion /exp
11. early rehabilitation /exp
12. early mobilization /exp
13. early motion /exp
14. sling free /exp
15. brace free /exp
16. 8 or 9 or 10 or 11 or 12 or 13 or 14 or 15
17. sling /exp
18. brace /exp
19. sling protection /exp
20. brace protection /exp
21. immobilization /exp
22. 17 or 18 or 19 or 20 or 21
23. controlled clinical trial /lim
24. randomized controlled trial /lim
25. systematic review /lim
26. meta analysis /lim
27. 23 or 24 or 25 or 26
28. 7 and (16 or 22)
29. 28 and 27

Web of Science (779 studies)

# 5 (#4 AND (#3 OR #2) AND #1) AND (English)

# 4 TS=(functional outcome OR PROM OR outcome)

# 3 TS=(immobilization OR brace OR protection OR sling)

# 2 TS=(early mobilization OR early rehabilitation OR early protocol OR mobilization OR rehabilitation)

# 1 TS=(Rotator cuff tear OR Shoulder Arthroscopy OR Supraspinatus tear OR Subscapularis tear OR biceps tendon tear OR labral tear OR shoulder instability OR Rotator Cuff repair)

Scopus (652 studies)

( functional AND outcome OR prom OR outcome ) AND ( immobilization OR brace OR protection OR sling OR early AND mobilization OR early AND rehabilitation OR early AND protocol OR mobilization ) AND ( rotator AND cuff AND tear OR shoulder AND arthroscopy OR supraspinatus AND tear OR subscapularis AND tear OR biceps AND tendon AND tear OR labral AND tear OR shoulder AND instability OR rotator AND cuff AND repair )

**Supplementary Figure 1.** : Risk of bias summary and risk of bias graph


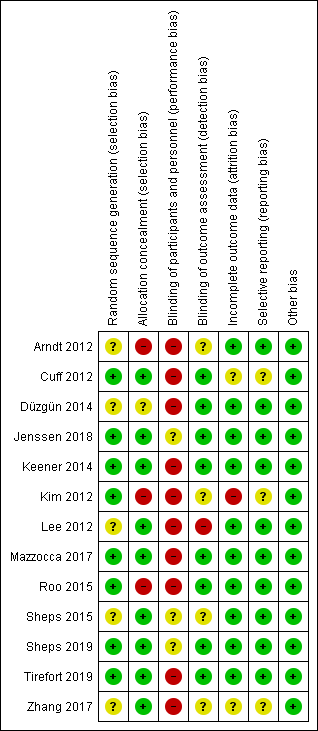

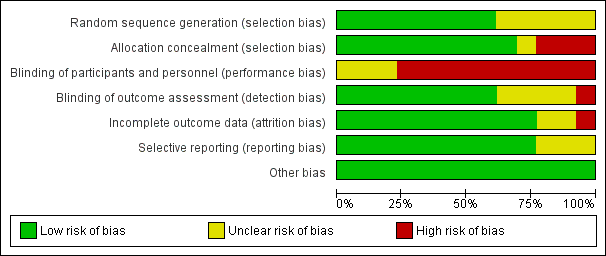


**Supplementary Figure 2~7.** : Funnel plot of outcomes

**EPM vs EAM**

***ROM***

Fig 2.1: anterior flexion final
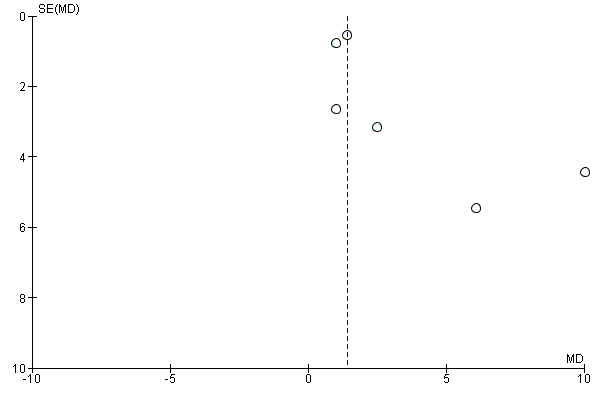


Fig 2.2: external rotation final
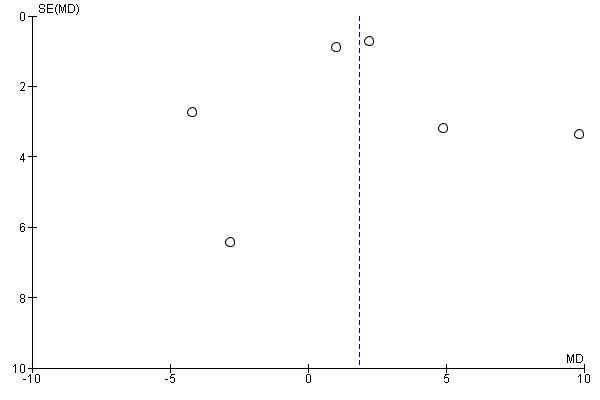


***Functional score***

Fig 3.1: Constant score final


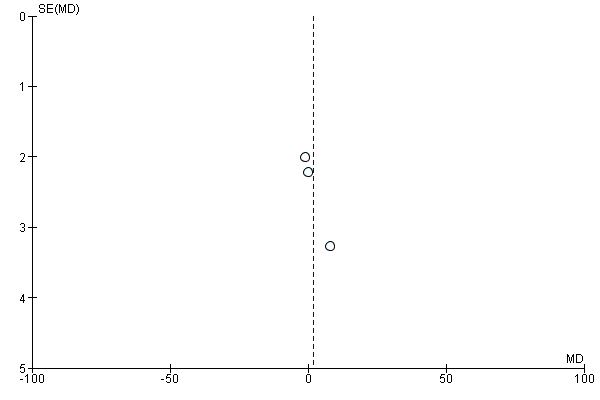


Fig 3.2: Simple Shoulder Test Score mid-term


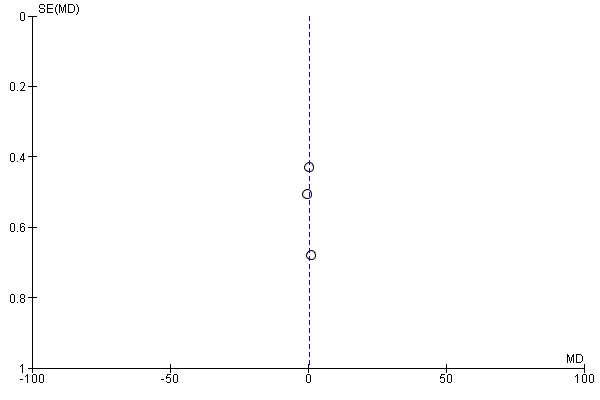


Fig 4: re-tear rate final


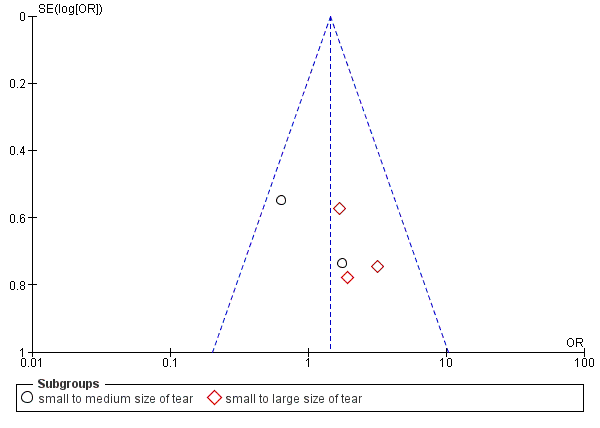


**EAM vs DAM**

***ROM***

Fig 5.1: anterior flexion final


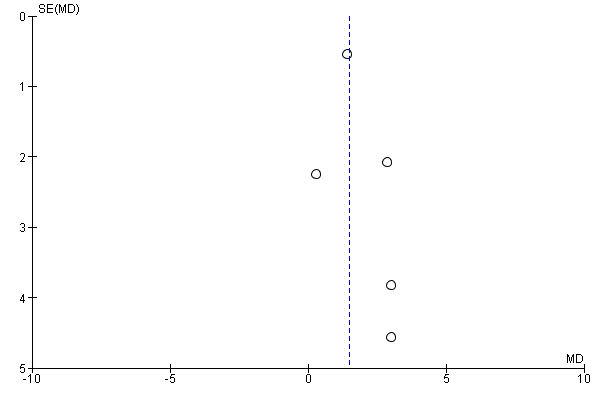


Fig 5.2: external rotation final


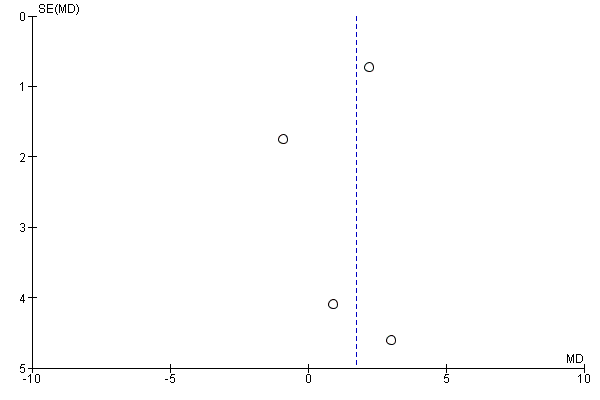


Fig 5.3: abduction final


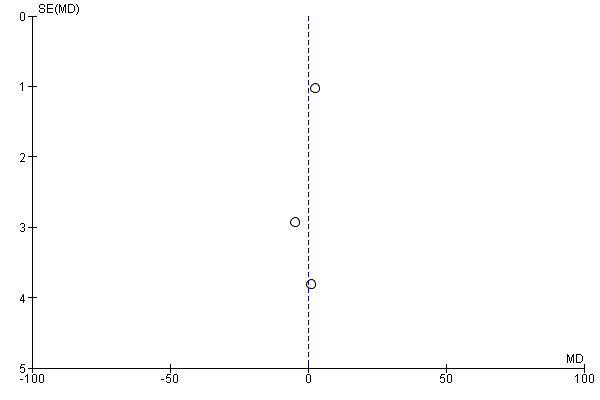


***Functional score***

Fig 6.1: Constant score final


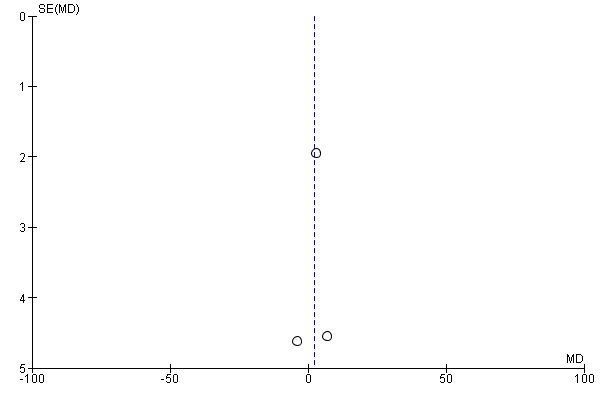


Fig 6.2 VAS preoperative


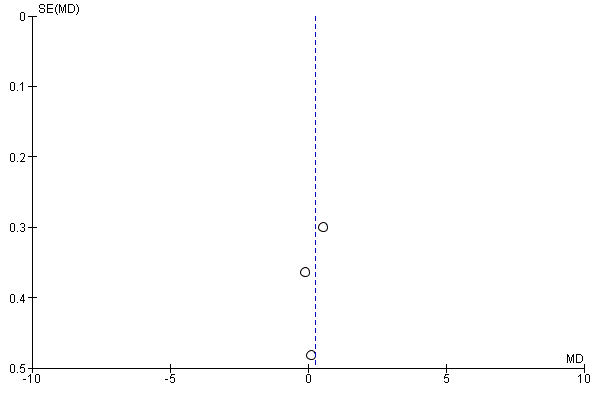


Fig 6.3 VAS final


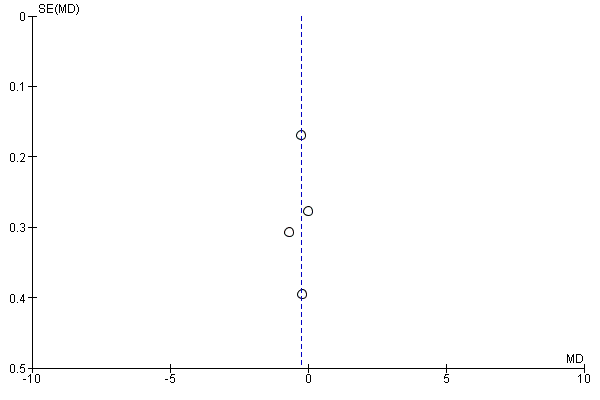


Fig 7: re-tear rate final


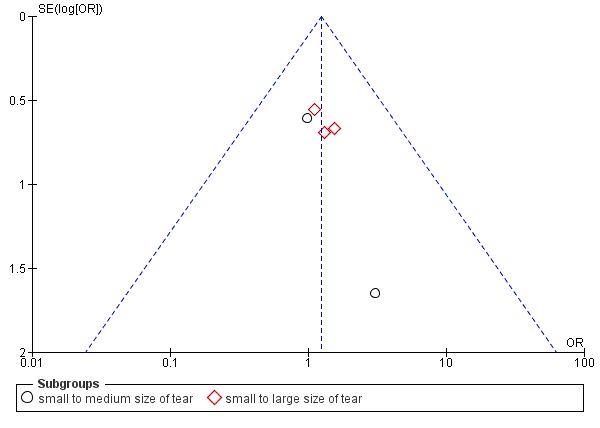


**Supplementary Figure 8~16.** : Forest plot of other outcomes

**DPM vs EPM**

***ROM***

Fig 8.1: anterior flexion preoperative
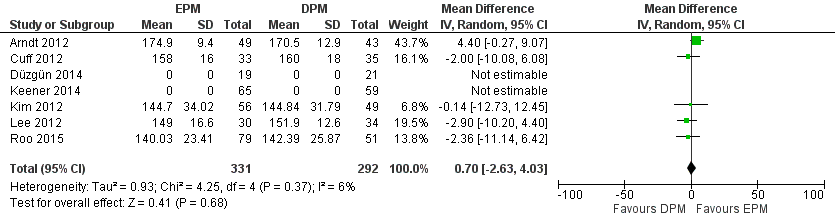


Fig 8.2: anterior flexion short term


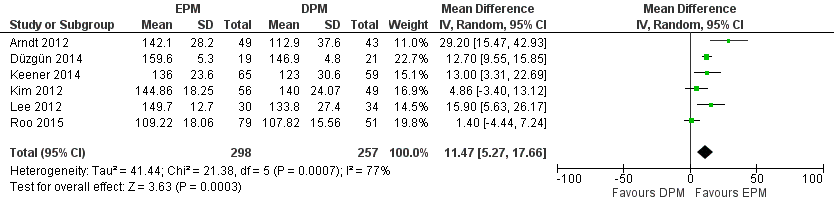


Fig 8.3: anterior flexion mid- term


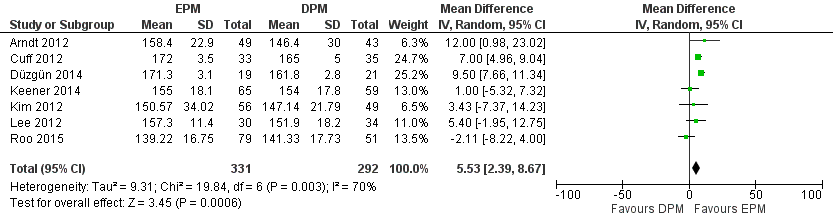


Fig 9.1: external rotation preoperative


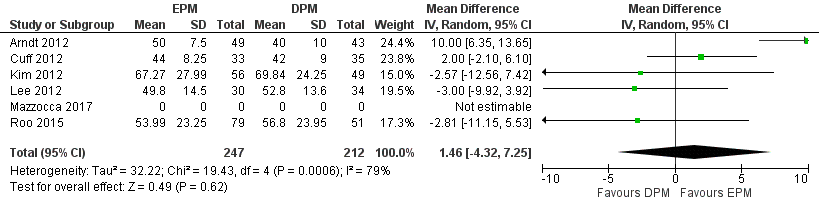


Fig 9.2: external rotation short term


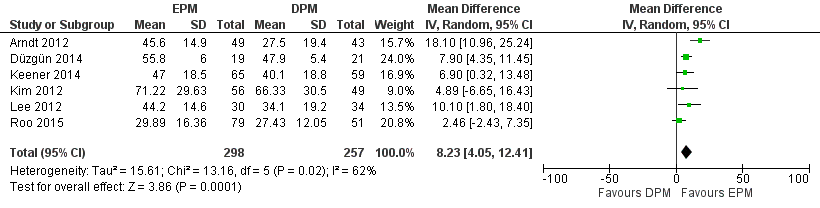


Fig 9.3: external rotation mid- term


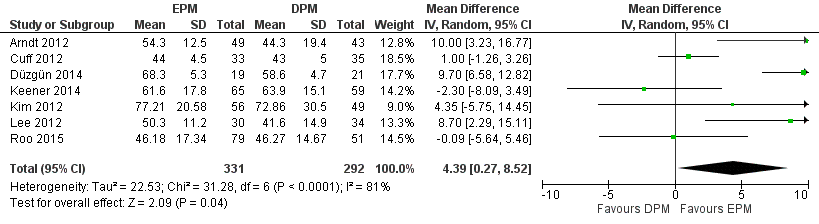


Fig 10.1: abduction preoperative


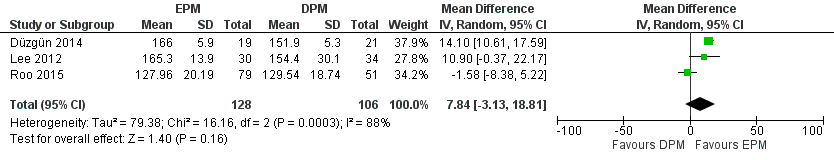


Fig 10.2: abduction short term


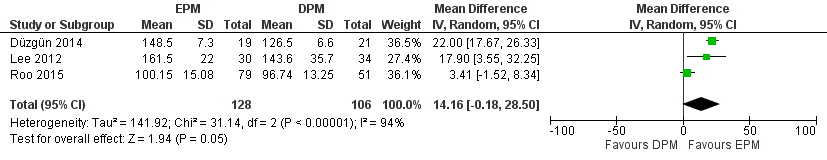


Fig 10.3: abduction mid- term


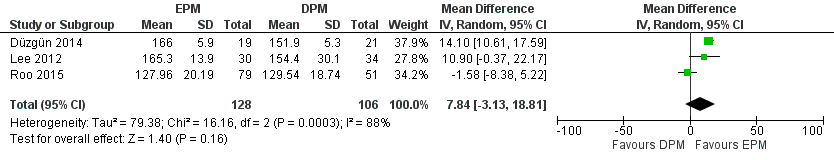


***Functional score***

Fig 11.1: Constant score mid-term
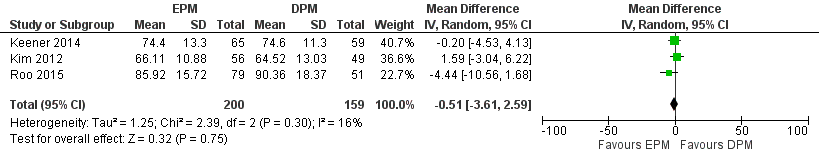


Fig 11.2: Constant score final


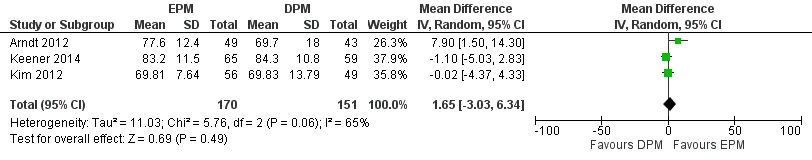


Fig 11.3: Simple shoulder test mid-term


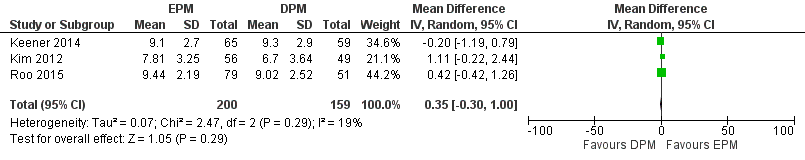


**DAM vs EAM**

***ROM***

Fig 12.1: anterior flexion preoperative


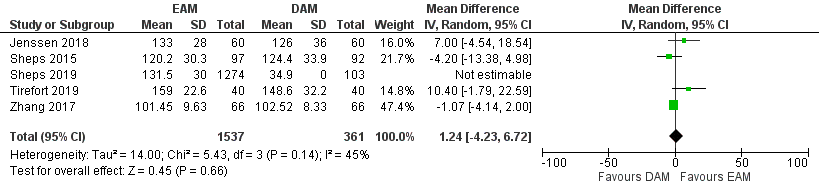


Fig 12.2: anterior flexion short term


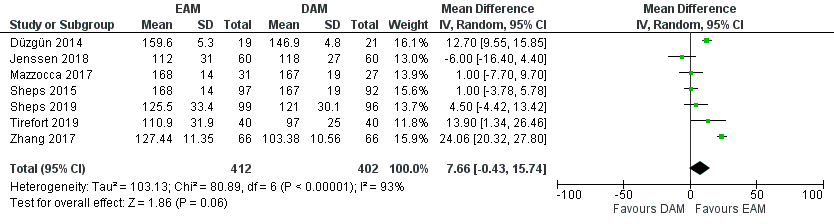


Fig 13.3: anterior flexion mid- term


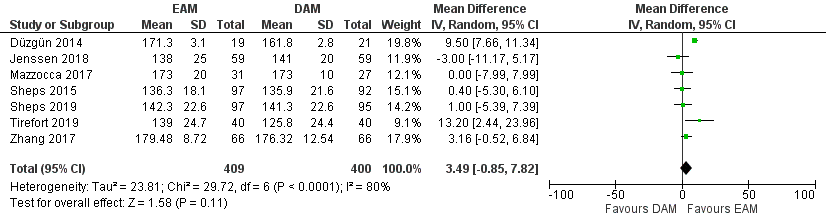


Fig 14.1: external rotation preoperative


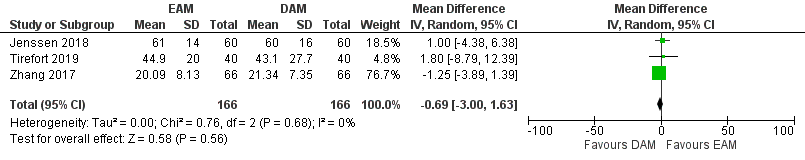


Fig 14.2: external rotation short term
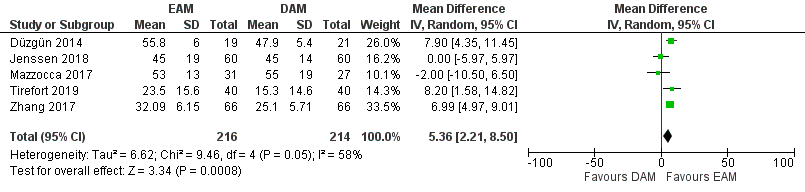


Fig 14.3: external rotation mid- term


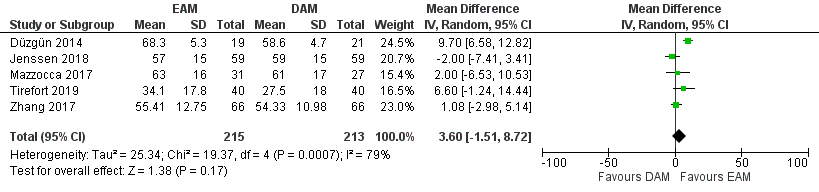


Fig 15.1: abduction preoperative


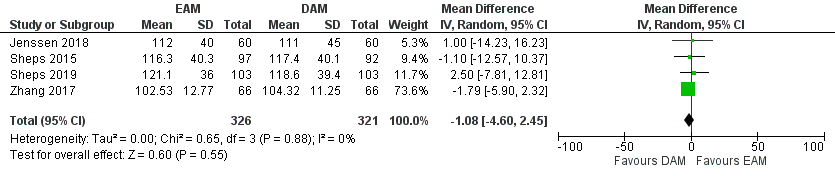


Fig 15.2: abduction short term


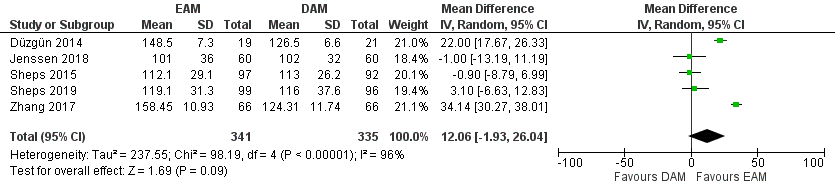


Fig 15.3: abduction mid- term


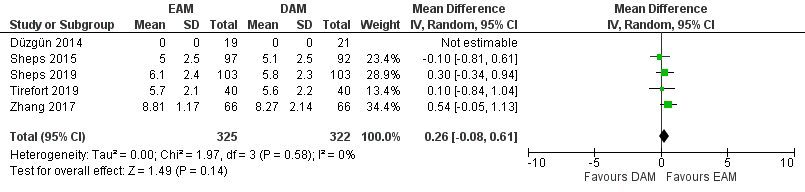


***Functional score***

Fig 16.1: Constant score final


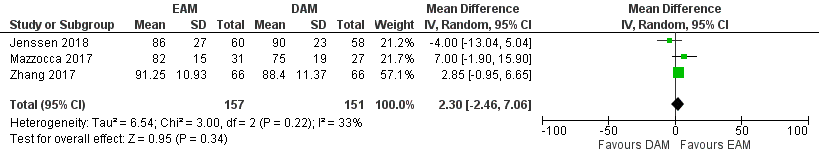


Fig 16.2: VAS preoperative


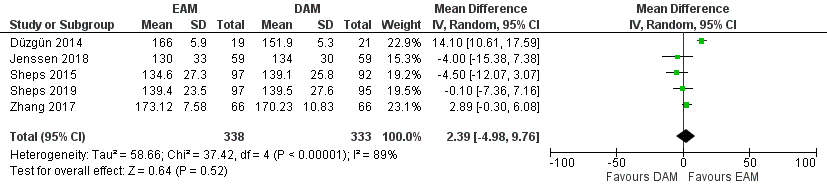


Fig 16.3: VAS short term


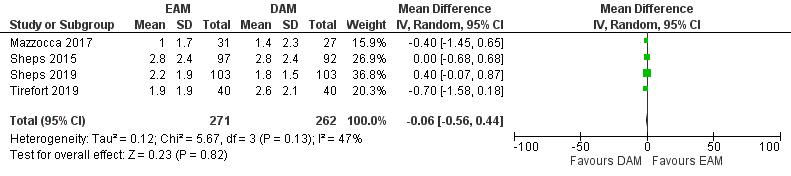


Fig 16.4: VAS mid-term


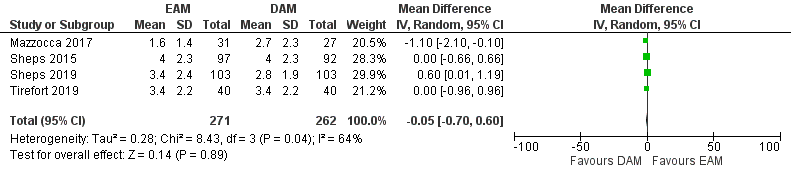


Fig 16.5: VAS final


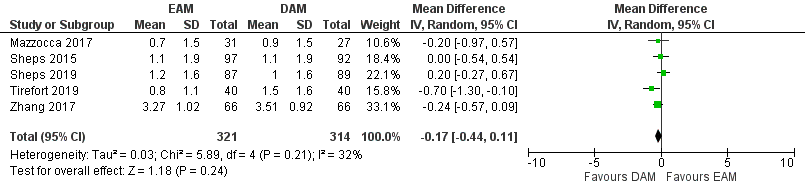


**Table 2: Grade-Assessment-of-Quality-of-Evidence**

|  | Outcome | Number of studies | Number of Participants | Risk of bias | Imprecision | Inconsistency | Indirectness | Publication bias | Relative Effect (95% Confidence Interval) | Confidence in Effect Estimate (Grade) |
| --- | --- | --- | --- | --- | --- | --- | --- | --- | --- | --- |
| EPM vs DPM | Anterior  flexion | 6 | 493 | Not serious | Not serious | Not Serious | Not serious | Serious | 1.40 (0.55-2.25) | Moderate |
|  | External  rotation | 6 | 493 | Not serious | Not serious | Serious | Not serious | Not serious | 1.86 (-0.53-4.25) | Moderate |
|  | Abduction | 2 | 104 | Not serious | Serious | Not serious | Not serious | Serious | 2.73 (0.74-4.71) | Low |
|  | Retear rate | 5 | 445 | Not serious | Not serious | Not serious | Not serious | Serious | 1.44 (0.83-2.52) | Moderate |
|  | CMS | 3 | 321 | Not serious | Serious | Serious | Not serious | Serious | 1.65 (-3.03- 6.34) | very low |
|  | SST | 3 | 359 | Not serious | Serious | Not serious | Not serious | Serious | 0.35 (-0.30, 1.00) | Low |
| EAM vs DAM | Anterior  flexion | 7 | 794 | Not serious | Not serious | Not serious | Not serious | Serious | 1.57 (0.62-2.52) | Moderate |
|  | External  rotation | 5 | 429 | Not serious | Not serious | Not serious | Not serious | Not serious | 1.59 (0.36-2.82) | High |
|  | Abduction | 5 | 656 | Not serious | Not serious | Not serious | Not serious | Not serious | 0.74 (-1.97-3.45) | High |
|  | Retear rate | 5 | 565 | Not serious | Not serious | Not serious | Not serious | Not serious | 1.24 (0.68-2.25) | High |
|  | CMS | 3 | 308 | Not serious | Serious | Not serious | Not serious | Serious | 2.30 (-2.46, 7.06) | Low |
|  | VAS | 5 | 635 | Not serious | Not serious | Not serious | Not serious | Not serious | -0.17 (-0.44, 0.11) | High |
